# Virtual morphometric method using seven cervical vertebrae for sex estimation on the Turkish population

**DOI:** 10.1007/s00414-021-02510-5

**Published:** 2021-02-05

**Authors:** Oguzhan Ekizoglu, Elif Hocaoglu, Ercan Inci, Gokce Karaman, Julieta Garcia-Donas, Elena Kranioti, Negahnaz Moghaddam, Silke Grabherr

**Affiliations:** 1 Department of Forensic Medicine, Tepecik Training and Research Hospital, Izmir, Turkey; 2 University Center of Legal Medicine Lausanne-Geneva, Geneva, Switzerland; 3 Department of Radiology, Bakirkoy Sadi Konuk Training and Research Hospital, Istanbul, Turkey; 4 Manisa Branch of Forensic Medicine, Council of Forensic Medicine, Manisa, Turkey; 5 Centre for Anatomy and Human Identification, School of Science and Engineering, University of Dundee, Dundee, Scotland DD1 5EH UK; 6 Department of Forensic Science, University of Crete, Heraklion, Crete Greece; 7 Unit of Forensic Imaging and Anthropology, University Center of Legal Medicine Lausanne-Geneva, Geneva, Switzerland; 8 Swiss Human Institute of Forensic Taphonomy, University Center of Legal Medicine Lausanne-Geneva, Geneva, Switzerland

**Keywords:** Sex estimation, Cervical vertebrae, Computed tomography, Virtual

## Abstract

Sex estimation from skeletal remains is crucial for the estimation of the biological profile of an individual. Although the most commonly used bones for means of sex estimation are the pelvis and the skull, research has shown that acceptable accuracy rates might be achieved by using other skeletal elements such as vertebrae. This study aims to contribute to the development of sex estimation standards from a Turkish population through the examination of CT scans from the seven cervical vertebrae. A total of 294 individuals were included in this study. The CT scans were obtained from patients attending the Bakirkoy Training and Research Hospital (Turkey) and the data was collected retrospectively by virtually taking measurements from each cervical vertebrae. The full database was divided into a training set (*N* = 210) and a validation set (*N* = 84) to test the fit of the models. Observer error was assessed through technical error of measurement and sex differences were explored using parametric and non-parametric approaches. Logistic regression was applied in order to explore different combinations of vertebral parameters. The results showed low intra- and inter-observer errors. All parameters presented statistically significant differences between the sexes and a total of 15 univariate and multivariate models were generated producing accuracies ranging from a minimum of 83.30% to a maximum of 91.40% for a model including three parameters collected from four vertebrae. This study presents a virtual method using cervical vertebrae for sex estimation on the Turkish population providing error rates comparable to other metric studies conducted on the postcranial skeleton. The presented results contribute not only to the development of population-specific standards but also to the generation of virtual methods that can be tested, validated, and further examined in future forensic cases.

## Introduction

Sex estimation through the examination of skeletal remains is one of the first steps in creating a reliable biological profile and plays a key role in terms of identification [1, 2]. In most cases, sex must be estimated before age, ancestry, and stature due to biological differences between males and females having an impact on the assessment of other pieces of biological information [2, 3]. Anthropological sex estimation consists of two main methodological approaches: morphological and metric analyses [2]. Morphological approaches are based on the visual evaluation of sexually dimorphic features and are mainly focused on the pelvis and skull, but also on the overall status including the differentiation of the robusticity of the bones and observations of various muscle marks [2]. On the other hand, metric methods are based on size differences between female and male individuals and typically use other postcranial elements along with the skull and the pelvis [2].

Metric approaches remain the most commonly used methods for sex estimation among forensic anthropologists [4]. Although the most reliable sex classification results are obtained from the analysis of the pelvis and skull [5], in situations such as natural and/or mass disasters, it may not always be possible to examine these skeletal elements due to animal activity, deliberate damage and skeletal disruption due to taphonomic processes. Jantz and Ousley [4] reported that postcranial measurements showed greater shape dimorphism than did cranial measurements, both individually and in combination. Thus, sex estimation may be required through the examination of other skeletal elements, as demonstrated by previous studies focusing on metatarsals, carpal bones, long bones, scapula, clavicle, patella and sternum [1]. In forensic investigations, methods used in sex estimation are expected to have an accuracy rate of more than 80% [3].

Metric methods use statistical analysis involving various approaches such as discriminant function analysis and logistic regression that provide equations to estimate sex in an unknown individual [6]. This makes it easier to assess quantitative outcomes obtained by osteometric approaches [1, 7]. With the development of molecular techniques, sex can be determined more reliably by identifying the presence or absence of the Y chromosome in the analysis performed from skeletal remains [2]. Molecular methods may be useful especially in juvenile skeletal remains, where osteological sex estimation methods present significant limitations [2]. Sexual dimorphism in the skeleton begins to develop with the release of sex hormones during puberty and becomes obvious around the age of 17 in many populations [2]. For this reason, many anthropologists consider sex estimation from the skeleton controversial before the age of 15, where molecular methods might assist in sex discrimination better than osteological sex biomarkers [2]. However, molecular methods entail a sophisticated analysis requiring high skills and expensive and advanced laboratory equipment [1]. Thus, they are considered complicated, costly, invasive, and time-consuming [1, 8]. The upper spine is one skeletal segment that remains to be further explored for sex estimation. However, sex estimation studies on the cervical vertebrae are limited and mostly focus on the axis and atlas or on the seventh cervical vertebra due to their atypical morphological structure and easier identification [9–14]. Research performed on the remaining cervical vertebrae in terms of sex estimation is thus limited [15], but accuracies over 80% have been reported by previous studies [9–16]. As the degree of sexual dimorphism and body proportions can differ between populations, and the most accurate vertebral sex markers may differ between geographical samples, population-specific studies are required to provide the most accurate outcomes [6].

At present, no research has been conducted on a Turkish population regarding sex estimation using a segment of the spine. Thus, our study aims to explore the possibility of developing population-specific standards for a Turkish sample by acquiring osteometric parameters through CT images of the seven cervical vertebrae and was the aim to provide alternatives in case the whole skeleton is not preserved.

## Materials and methods

### Subjects

This study was conducted at the Bakirkoy Training and Research Hospital, Turkey. Medical records and computed tomography (CT) images of 294 patients (146 male and 148 female) admitted to the various clinics of the hospital from 2015 to 2020 with diagnoses of trauma were retrospectively evaluated. Age ranges from 18 to 87 years and mean ages are 39.27 ± 14.83 for males and 44.76 ± 12.94 for females. The ethics protocol for this study was obtained under Ethics clearance number 2020/4-4. Patients with any pathology of the cervical vertebrae (e.g., tumors, fractures, infections, surgical fixations) and other neoplastic disorders were excluded, as well as those with CT images with motion artifacts.

### Image acquisition

All cervical CT examinations were performed using a 128 slice multidetector CT scanner (Siemens Medical Solutions, Erlangen, Germany). A routine cervical CT protocol was followed with a 1-mm slice thickness in the supine position. Tube voltage was 120 kV; effective mAs was adjusted by Siemens “SAFIRE reconstruction software.” Gantry rotation was 0.5 s, collimation was 0.6 mm, and the pitch was 1.2 mm. All patients underwent imaging from the base of cranium to thoracic inlet.

### Image analysis

Three measurements were collected from each of the seven cervical vertebrae: maximum cervical vertebral body height (CHT), cervical anterior-posterior diameter (CAP), and cervical transverse diameter (CTR); CHT was not collected for the first cervical vertebra, as this vertebra does not have a centrum. In total, 20 measurements were taken for the purposes of this study.

**Fig. 1.**
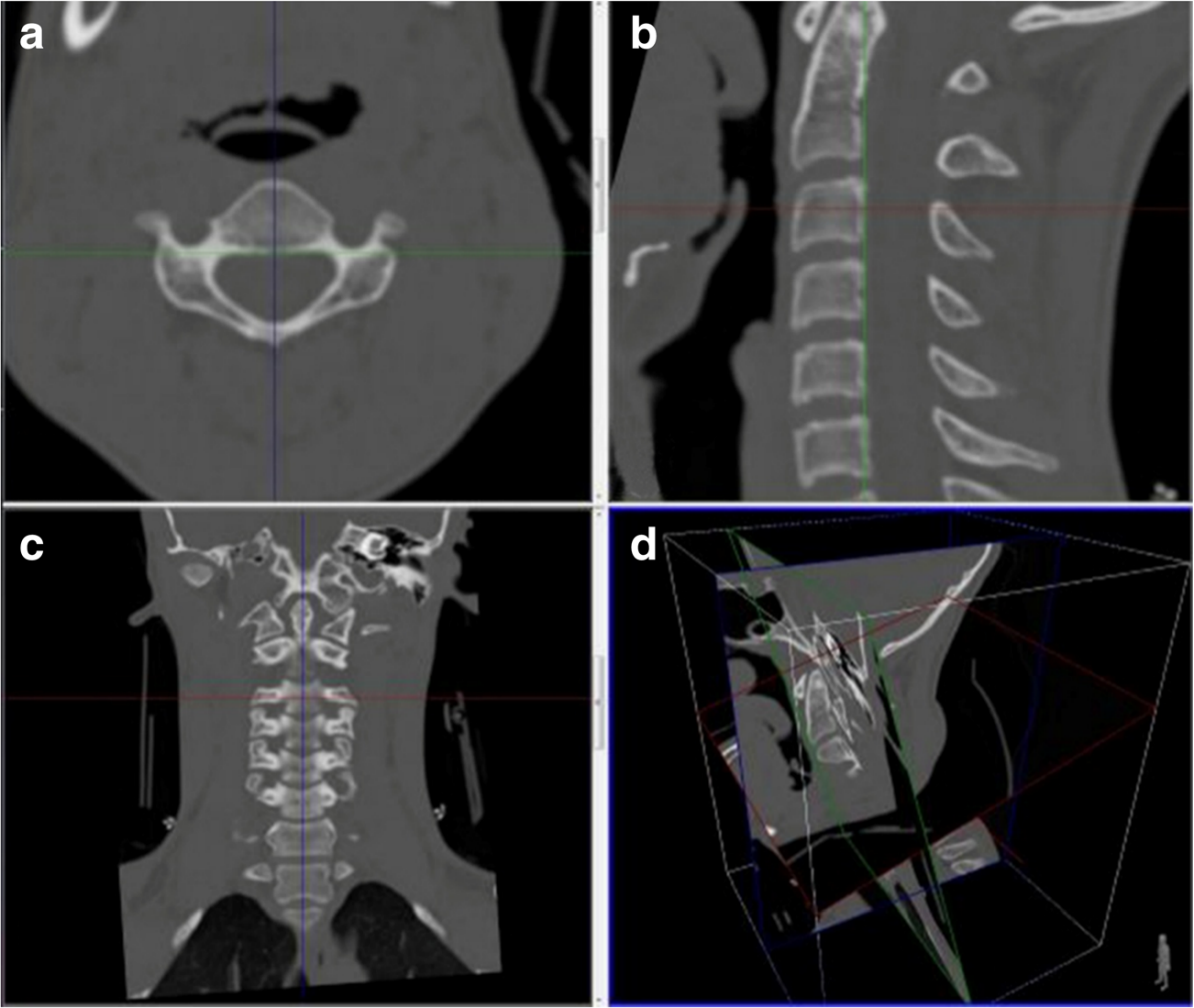
Determination of the CAP and CTR with axial (**a**), sagittal (**b**), coronal (**c**), and three-dimensional projections of section plans (**d**) reconstructed CT images

**Fig. 2:**
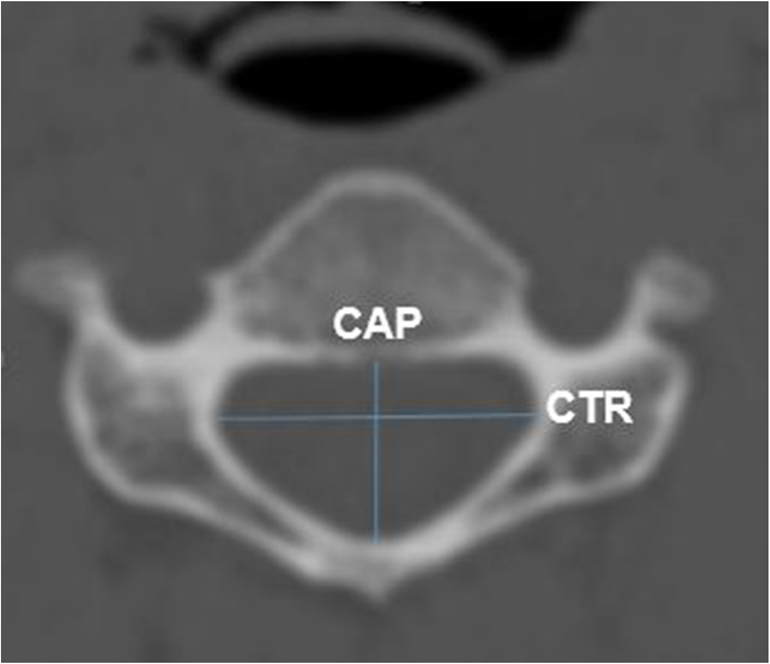
Measurements of CAP and CTR on axial CT image

**Fig. 3:**
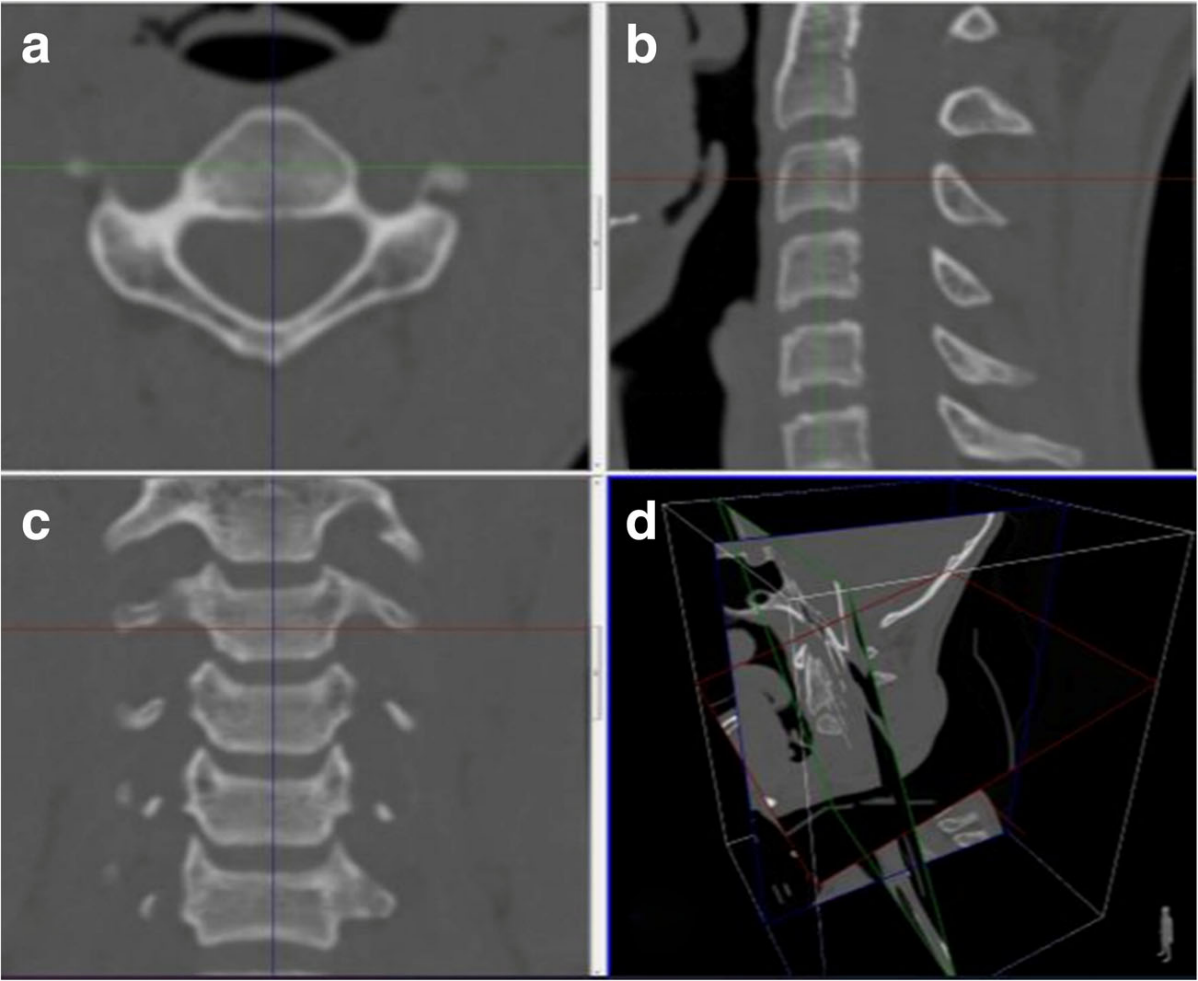
Determination of the CHT with axial (**a**), sagittal (**b**), coronal (**c**), and three-dimensional projections of section plans (**d**) reconstructed CT images

**Fig. 4:**
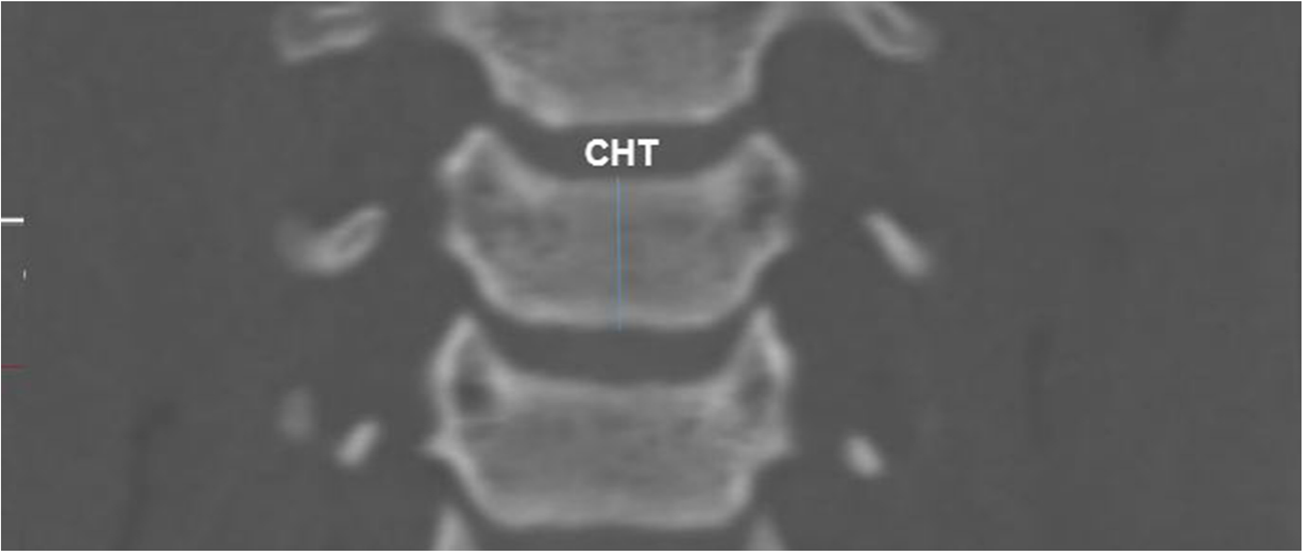
Measurements of CHT on coronal CT image

The measurement parameters we have determined for the spinal canal and vertebral corpus in each vertebra at the level of C1–C7 vertebrae are explained below and showed Figs. 1, 2, 3 and 4.

First, coronal and sagittal reformatted images were created from axial source images.

Secondly, for spinal canal measurement at the level of each cervical vertebra corpus, in the coronal reformat images, the midline was determined with reference to the odontoid process (Fig. 1c, blue reference line). Then, in the sagittal images, the line that fits the vertebral corpus posterior contour to be measured for each vertebra was chosen as a reference (Fig. 1b, green reference line). After these two arrangements, the section level where the spinal canal can be viewed continuously in the axial images was chosen (Fig. 1a). In this section, spinal canal anterior-posterior diameter and medial-lateral diameter measurements were made (Fig. 2).

Thirdly, for height measurement at the level of each cervical vertebral corpus; In the axial source images, the anterior-posterior diameter and medial-lateral diameter of the vertebral corpus were determined, and the reference lines in the axial source and sagittal reformat images were localized to this point (Fig. 3a and b). As a result, vertebral corpus height was determined by measuring between the superior-inferior endplates in coronal reformatted images (Fig. 4)

All measurements were performed by two independent observers. Both were experienced radiologists, fully trained in musculoskeletal radiology and forensic imaging. Each had 10 years of experience in the field of forensic radiology and completed different virtual anthropology studies. Observers have also studied published articles on the cervical vertebra and were trained in measurement techniques with an experienced forensic pathologist.

### Statistical analysis

SPSS 22.0 (IBM Corporation, Armonk, NY, USA) program was used for statistical analyses.

Observer error was measured through technical error of measurement (TEM) analysis [17]. Intra-observer error was assessed on 40 random CT images with a two-week interval between the first and the second observation. Inter-observer error was performed on the same 40 CT images by a second observer.

From the total sample set (*N* = 294), 70% (*N* = 210) was used for the generation of the logistic regression (LR) equations and 30% (*N* = 84) was used as a validation set with the main statistical analysis being performed using the developmental set. First, the dataset was examined to assess normality, outliers, skewness and kurtosis. Secondly, all the parameters were examined to explore whether statistically significant differences exist between males and females.

Binary logistic regression (LR) was the statistical approach used in this study, as the data did not meet the assumptions for discriminant function analysis. Moreover, LR is more flexible as it is less dependent to outliers and generally more tolerant to co-linearity of predictors [9]. LR allows the discrimination between groups following the following formula:1where *L* is the logit or log-odd, *C* is the constant and *b* and *X* are the regression coefficient and the measurement, respectively. The cutting point is set at 0.5 with males scoring over this value. LR modeling was performed considering the identification of vertebrae number, the combination of variables and the recovery and preservation of a partial or full set of cervical vertebrae. Models were evaluated based on LR assumption and overfitting considering the possibility of including the fewer number of variables (reducing measurement error) and achieving the highest correct classification.

LR modeling was first run by each independent vertebra including manually all three parameters in the model (CHT, CAP, and CTR). The second step in the generation of the most optimal models consists of the combination of two consecutive cervical vertebrae (e.g. C2 and C3, C3 and C4, and so on) in an attempt to provide equations for vertebrae that can be identified based on the articulation with the consecutive one. Note that some model modifications were made after the observation of model fit indicators, and in some instances, variables no contributing to the model were removed in order to improve the fit. LR was also run on three consecutive vertebrae although the results did not show any improvement as compared to the two consecutive vertebrae, and therefore, these models are not reported. The third step was to use C1 to C7 to create a single equation contemplating the scenario in which the full set of cervical vertebrae is intact and present. Note that even if the total of number of parameters assessed in this study is 20, a maximum of 10 variables is recommended due to statistical constrains based on the sample size [18]. As CHT was reported to be the most sexually dimorphic parameter, stepwise forward LR was performed on all CHT measurement for all cervical vertebrae, followed by the inclusion of CTR and CAP block of measurements, separately. In the last step of the statistical analysis, stepwise forward LR modeling was used including all 20 measurements to identify the most optimal combination of variables. Only LR equations that achieved overall correct sex classification over 80% are reported here. The selected models were then cross-validated using the training set to test the stability of the models with those that hold within 10% classification accuracy of the original primary sample being considered valuable models [19].

The individuals included in this study were divided into five age cohorts (18–29, 30–39, 40–49, 50–59, and 60 years old and above). Defined age groups were tested for normality using Shapiro-Wilk test. Normality was violated on all occasions and the null hypothesis of equal covariance matrices was rejected, and thus, a non-parametric test (Kruskal-Wallis test) to explore if there are any statistically significant differences between age groups for cervical measurements at significance level of *p* < 0.05.

## Results

This project includes 41,160 metric data obtained from CT images of 2058 vertebrae of 294 adults from contemporary Turkish population.

Observer error results are shown in Table 1. Both intra and inter-observer errors fell within the limits of acceptance as seen by the low values for rTEM and *R*. The only two parameters showing more than 10% of the variance related to variability between subject scores were reported for C3HT and C5AP (*R* = 0.89).

**Table 1:** TEM, rTEM, and *R* intra- and inter-observer error for the variables included in the study

	Intra-observer (*n* = 40)			Inter-observer (*n* = 40)		
	TEM	rTem	*R*	TEM	rTem	*R*
C2HT	0.379	1.056	0.98	0.409	1.280	0.96
C3HT	0.317	2.342	0.95	0.351	2.956	0.89
C4HT	0.318	2.450	0.95	0.257	2.218	0.94
C5HT	0.373	2.945	0.92	0.330	2.897	0.90
C6HT	0.254	2.028	0.96	0.245	2.164	0.92
C7HT	0.352	2.508	0.94	0.268	2.101	0.93
C1AP	0.392	1.236	0.96	0.253	0.856	0.96
C2AP	0.346	2.147	0.94	0.243	1.600	0.97
C3AP	0.315	2.155	0.95	0.366	2.680	0.90
C4AP	0.357	2.568	0.93	0.265	2.058	0.95
C5AP	0.256	1.860	0.96	0.343	2.677	0.89
C6AP	0.361	2.659	0.92	0.246	1.963	0.95
C7AP	0.239	1.720	0.97	0.252	1.907	0.93
C1TR	0.254	0.886	0.99	0.229	0.842	0.98
C2TR	0.366	1.501	0.96	0.345	1.514	0.90
C3TR	0.343	1.463	0.97	0.280	1.235	0.95
C4TR	0.312	1.294	0.97	0.263	1.141	0.96
C5TR	0.285	1.145	0.98	0.223	0.944	0.98
C6TR	0.257	1.038	0.96	0.274	1.163	0.97
C7TR	0.307	1.241	0.95	0.313	1.331	0.97

*TEM*, technical error measurement; *rTEM*, relative TEM; *n*, number of individuals; *C*, cervical; *1–7*, from first to seventh vertebrae; *HT*, maximum body height; *TR*, foramen transverse diameter; *AP*, foramen anterior-posterior diameter

Demographic data for males and females for both the developmental set (106 males and 104 females) and the training sets (40 males and 44 females) are presented below (Table 2).

**Table 2:** Descriptive statistics for training set and sexual dimorphism indicator (SDI) and descriptive statistics for the validation set

Training set							Validation set			
	Sex	Minimum	Maximum	Mean	SD	SDI %	Minimum	Maximum	Mean	SD
C2HT	M	31.82	38.93	36.33	1.97	10.5	31.82	38.93	36.33	1.97
	F	27.52	37.11	32.49	1.97		28.83	35.62	32.21	1.61
C3HT	M	10.94	16.15	13.62	1.27	12.16	10.94	16.15	13.62	1.27
	F	9.42	14.49	11.96	1.05		9.95	14.88	11.91	1.02
C4HT	M	11.12	16.17	13.2	1.17	12.18	11.12	16.17	13.2	1.17
	F	9.25	14.05	11.59	0.89		9.95	14.62	11.58	0.95
C5HT	M	11.09	16.84	13.14	1.16	13.26	11.09	16.84	13.14	1.16
	F	8.87	13.82	11.4	0.97		9.22	13.55	11.19	1.06
C6HT	M	10.39	16.32	12.89	1.25	11.8	10.39	16.32	12.89	1.25
	F	9.13	14.03	11.36	0.9		9.3	13.15	11.29	0.85
C7HT	M	12.68	18.36	14.39	1.33	12.42	12.68	18.36	14.39	1.33
	F	9.97	14.73	12.61	0.93		10.49	14.05	12.65	0.82
C1AP	M	28.71	36.23	32.48	2.12	8.28	28.71	36.23	32.48	2.12
	F	25.39	35.81	29.79	1.76		26.3	34.83	29.88	1.73
C2AP	M	12.19	20.59	16.77	1.77	7.4	12.19	20.59	16.77	1.77
	F	12.44	18.6	15.53	1.12		13.13	18.08	15.7	1.39
C3AP	M	10.11	18.07	14.65	1.63	5.46	10.11	18.07	14.65	1.63
	F	11.02	17.13	13.85	1.06		12.28	15.62	13.79	0.93
C4AP	M	8.55	16.71	14.02	1.52	4.7	8.55	16.71	14.02	1.52
	F	10.71	16.89	13.36	1.08		10.93	15.37	13.19	0.98
C5AP	M	9.38	16.85	14.05	1.54	5.8	9.38	16.85	14.05	1.54
	F	10.73	16	13.22	1.08		11.15	15.08	13.1	0.92
C6AP	M	8.69	17.09	14.07	1.52	7.9	8.69	17.09	14.07	1.52
	F	10.73	15.96	12.96	1.08		10.88	15.56	12.93	1
C7AP	M	11.4	19.21	14.48	1.47	8.4	11.4	19.21	14.48	1.47
	F	10.67	15.63	13.25	1.12		10.42	15.25	13.31	1.04
C1TR	M	22.57	33.35	28.65	2.35	4.44	22.57	33.35	28.65	2.35
	F	23.22	32.66	27.38	1.85		23.26	31.82	27.55	2.07
C2TR	M	20.41	27.5	24.55	1.73	7.2	20.41	27.5	24.55	1.73
	F	19.13	26.29	22.77	1.31		20.1	26.93	23.09	1.48
C3TR	M	19.69	26.9	23.94	1.69	7	19.69	26.9	23.94	1.69
	F	19.62	26.08	22.26	1.29		19.35	26.15	22.55	1.44
C4TR	M	20.39	28.18	24.33	1.64	6.29	20.39	28.18	24.33	1.64
	F	19.42	26.42	22.8	1.39		20.12	28.51	23.16	1.47
C5TR	M	21.44	28.76	24.82	1.54	5.7	21.44	28.76	24.82	1.54
	F	19.59	27.41	23.39	1.47		20.85	29.82	23.9	1.64
C6TR	M	21.44	30.68	25.1	1.84	6.04	21.44	30.68	25.1	1.84
	F	19.88	26.83	23.58	1.51		20.66	28.76	23.83	1.5
C7TR	M	21.82	27.59	25.07	1.45	6.8	21.82	27.59	25.07	1.45
	F	19.15	27.1	23.34	1.55		20.08	27.2	23.24	1.79

*C*, cervical; *1–7*, from first to seventh vertebrae; *HT*, maximum body height; *TR*, foramen transverse diameter; *AP*, foramen anterior-posterior diameter; *M*, male; *F*, female; *n*, number; *SD*, standard deviation; *SDI*, sexual dimorphism indicator

A sexual dimorphism indicator (SDI) per measurement was calculated following Gama et al. [9]. If the index is higher than 10%, the parameters are considered to be strongly sexually dimorphic: SDI = ((Males mean − Females mean) / Males mean)*100.

Regarding the assessment of normality, only two parameters, C4HT and C1AP, were non-normally distributed as indicated by Shapiro-Wilk test (*p* value < 0.05). Thus, those measurements were subject to non-parametric statistical tests.

To examine differences in cervical measurements between males and females, an independent sample *t* test or Welch test were performed based on the results provided by Levene’s test of equality of variances, while the non-parametric equivalent (Mann-Whitney *U* test) was performed on C4HT and C1AP. All normally distributed parameters demonstrated statistically significant differences between the sexes with all *p* values being less than 0.001 (Table 3). Additionally, Mann-Whitney *U* test indicated statistically significant differences for C4HT and C1AP (*p* < .0001, *z* = − 9.84 and *z* = − 7.75, respectively).

**Table 3:** Independent *t* test results for sex differences for the cervical measurements

	*t*	Mean difference	SE	95% CI	
				Lower	Upper
C2HT	13.84	3.871	0.280	3.320	4.423
C3HT	12.19	1.837	0.151	1.540	2.134
C5HT	12.15	1.723	0.142	1.444	2.003
C6HT	12.20	1.626	0.133	1.363	1.889
C7HT	12.81	1.978	0.154	1.674	2.283
C2AP	5.54	1.001	0.181	0.645	1.358
C3AP	5.57	0.977	0.176	0.631	1.323
C4AP	4.39	0.725	0.165	0.399	1.050
C5AP	5.18	0.874	0.169	0.541	1.206
C6AP	5.54	0.965	0.174	0.622	1.309
C7AP	6.02	1.063	0.176	0.715	1.411
C1TR	5.39	1.618	0.300	1.026	2.210
C2TR	7.97	1.580	0.198	1.189	1.971
C3TR	6.86	1.275	0.186	0.909	1.642
C4TR	7.12	1.413	0.198	1.022	1.804
C5TR	6.84	1.461	0.214	1.040	1.882
C6TR	7.44	1.557	0.209	1.145	1.970
C7TR	7.52	1.648	0.219	1.215	2.080

*All measurements were statistically significant at *p* value < 0.001

*SE*, standard error; *CI*, confidence interval

Table 4 shows the results for LR modeling by each independent vertebra inserting manually all three parameters in the model (CHT, CAP, and CTR). All the LR equations generated using parameters from each independent vertebra demonstrated statistically significant models in comparison to the null model. Note that model M2 does not include all three parameters because a better-fit model was created by the exclusion of those parameters that did not contribute significantly. The percentage of correct classification ranges from 83.8% for M5 up to a maximum of 87.6% for M2 (refer to Table 7). Note that cervical 1 is not reported as the percentage of correct classification was lower than 80% (75% accuracy).

**Table 4:** LR (logistic regression) by independent vertebrae

Model	Variables	*B*	SE	Wald	Sig.	Exp *B*
M2_Cervical 2	C2HT	1.03	0.145	50.502	0.000	2.800
	C2AP	0.779	0.193	16.291	0.000	2.179
	Constant	− 47.77	6.626	51.98	0.000	0.000
M3_Cervical 3	C3HT	1.545	0.237	42.578	0.000	4.689
	C3AP	0.727	0.178	16.586	0.000	2.068
	C3TR	0.338	0.155	4.715	0.030	1.402
	Constant	− 37.919	5.567	46.389	0.000	0.000
M4_Cervical 4	C4HT	1.68	0.25	45.263	0.000	5.367
	C4AP	0.66	0.185	12.703	0.000	1.936
	C4TR	0.327	0.156	4.406	0.036	1.387
	Constant	− 37.645	5.402	48.564	0.000	0.000
M5_Cervical 5	C5HT	1.7	0.255	44.419	0.000	5.476
	C5AP	0.762	0.179	18.151	0.000	2.144
	C5TR	0.373	0.146	6.543	0.011	1.452
	Constant	− 40.114	5.599	51.333	0.000	0.000
M6_Cervical 6	C6HT	1.67	0.254	43.104	0.000	5.312
	C6AP	0.577	0.176	10.709	0.001	1.780
	C6TR	0.52	0.151	11.893	0.001	1.681
	Constant	− 40.61	5.793	49.15	0.000	0.000
M7_Cervical 7	C7HT	1.719	0.276	38.75	0.000	5.576
	C7AP	0.413	0.183	5.11	0.024	1.511
	C7TR	0.526	0.145	13.169	0.000	1.693
	Constant	− 41.431	5.93	48.821	0.000	0.000

*SE*, standard error; *Sig*, significance

Measurements from two consecutive vertebrae were combined and LR models were generated (Table 5). The resulting models were statistically significant and correctly classified individuals with percentages ranging from 86.70 to 88.10 (refer to Table 6).

**Table 5:** LR (logistic regression) equations by two consecutive vertebrae

Model	Variables	*B*	SE	Wald	Sig.	Exp *B*
M8_C1 and C2	C1AP	0.403	0.152	7.017	0.008	1.496
	C2AP	0.525	0.204	6.627	0.010	1.690
	C2HT	0.982	0.15	42.914	0.000	2.669
	Constant	− 54.435	7.862	47.938	0.000	0.000
M9_C2 and C3	C2HT	0.851	0.161	27.845	0.000	2.342
	C3HT	0.963	0.26	13.675	0.000	2.619
	C3AP	0.909	0.224	16.544	0.000	2.483
	Constant	− 54.592	7.731	49.867	0.000	0.000
M10_C3 and C4	C3HT	0.831	0.306	7.383	0.007	2.295
	C3AP	0.781	0.18	18.907	0.000	2.184
	C4HT	1.132	0.318	12.632	0.000	3.101
	Constant	− 35.895	4.939	52.812	0.000	0.000
M11_C4 and C5	C4HT	1.027	0.337	9.258	0.002	2.792
	C5HT	0.939	0.345	7.397	0.007	2.558
	C5AP	0.793	0.19	17.33	0.000	2.209
	C5TR	0.312	0.152	4.223	0.040	1.366
	Constant	− 42.539	5.98	50.606	0.000	0.000
M12_C5 and C6	C5HT	1.059	0.314	11.375	0.001	2.883
	C6HT	1.007	0.33	9.327	0.002	2.736
	C6TR	0.457	0.161	8.098	0.004	1.579
	C5AP	0.72	0.186	15.061	0.000	2.054
	Constant	− 46.071	6.557	49.366	0.000	0.000
M13_C6 and C7	C6HT	0.78	0.318	6.015	0.014	2.181
	C7HT	1.236	0.328	14.204	0.000	3.443
	C6AP	0.558	0.194	8.313	0.004	1.747
	C7TR	0.51	0.15	11.56	0.001	1.665
	Constant	− 45.783	6.438	50.578	0.000	0.000

*SE*, standard error; *Sig*, significance

**Table 6:** Forward LR (logistic regression) using CHT, CTR, and CAP sets of parameters

Model	Variables	*B*	SE	Wald	Sig.	Exp *B*
M14_CHT	C2HT	0.62	0.15	17.207	0.000	1.859
	C4HT	0.655	0.26	6.354	0.012	1.924
	C7HT	0.759	0.285	7.096	0.008	2.137
	Constant	− 39.689	5.49	52.269	0.000	0.000
M15_CHT and CTR	C2HT	0.581	0.166	12.298	0.000	1.788
	C4HT	0.648	0.278	5.455	0.020	1.912
	C7HT	0.804	0.318	6.404	0.011	2.235
	C7TR	0.507	0.162	9.814	0.002	1.660
	Constant	− 50.947	7.55	45.53	0.000	0.000
M16_ALL stepwise	C2HT	0.705	0.171	16.991	0.000	2.024
	C3HT	0.633	0.295	4.595	0.032	1.883
	C6HT	0.884	0.34	6.744	0.009	2.420
	C1AP	0.389	0.18	4.648	0.031	1.475
	C3AP	0.779	0.237	10.849	0.001	2.180
	Constant	− 66.122	9.991	43.796	0.000	0.000

*SE*, standard error; *Sig*, significance; *C*, cervical; *HT*, maximum body height; *TR*, foramen transverse diameter; *AP*, foramen anterior-posterior diameter

Stepwise LR was performed by combining first all set of the most dimorphic parameters (CHT) and then adding separately CTR and CAP cervical measurement sets. As seen in Table 6, both M14 and M15 include cervical 2, 4 and 7. For the model created by the inclusion of all 20 parameters, four different vertebrae are necessary in order to use this equation including five measurements. This last model produced the highest percentage of correct classification both for original and cross-validated accuracies (Table 7).

**Table 7:** Original and cross-validated correct classification of all generated models for males, females, and total samples

			% correct classification	
		Sex	Training set	Validation set
C2	M2	M	87.50	82.50
		F	87.70	95.40
		Total	87.60	88.00
C3	M3	M	85.60	62.50
		F	84.00	87.50
		Total	84.80	76.20
C4	M4	M	86.50	65.00
		F	84.90	90.90
		Total	85.70	83.30
C5	M5	M	84.60	80.00
		F	83.00	86.40
		Total	83.80	82.14
C6	M6	M	85.60	72.50
		F	88.70	90.90
		Total	87.10	82.14
C7	M7	M	89.40	82.50
		F	84.00	90.90
		Total	86.70	86.90
C1+C2	M8	M	88.50	82.50
		F	85.80	95.45
		Total	87.10	89.20
C2+C3	M9	M	90.40	85.00
		F	85.80	97.70
		Total	88.10	91.60
C3+C4	M10	M	89.40	72.50
		F	86.80	95.45
		Total	88.10	84.50
C4+C5	M11	M	89.40	80.00
		F	86.80	88.63
		Total	88.10	84.50
C5+C6	M12	M	87.50	80.00
		F	85.80	93.20
		Total	86.70	85.70
C6+C7	M13	M	90.40	85.00
		F	84.90	95.45
		Total	87.60	90.50
CHT	M14	M	88.50	80.00
		F	86.80	97.70
		Total	87.60	89.30
CHT+TR	M15	M	92.30	90.00
		F	87.70	90.90
		Total	90.00	90.46
Stepwise all parameters	M16	M	92.30	85.00
		F	90.60	93.20
		Total	91.40	89.30

*M*, male; *F*, female

Regarding the statistical analysis for each measurement according to age groups, a significant difference was observed for C5AP (*p* = 0.040) and C4TR (*p* = 0.048) for males and C3HT (*p* = 0.000), C3AP (*p* = 0.038), C6AP (*p* = 0.005), C3TR (*p* = 0.003), and C4TR (*p* = 0.025) for females.

## Discussion

The current study examined the seven virtually reconstructed cervical vertebrae to establish an accurate sex estimation method for Turkish population. Morphometrically, 20 linear parameters of the seven cervical vertebrae were measured on CT images, and their predictive accuracy ranged from 83.30% to 91.40% as a result of logistic regression equations.

The cervical spine consists of three atypical segments (C1, C2, and C7) and four typical features (C3–C6). The vertebral body consists of several projections used for articulation with the vertebral arch [20]. The first vertebra (C1) is the largest, annular in shape and lacking a vertebral body and spinous process [20]. The second cervical vertebra (C2) includes an odontoid process extending from the vertebral body [20]. The seventh cervical vertebra (C7) presents a prominent spinous process, known as the vertebrae prominence, permitting differentiation from the third to sixth cervical elements [20]. The morphological characteristics of the cervical vertebrae make it easy to create anatomical sequences among all skeletal elements, including consecutive vertebrae [15]. The vertebral body provides strength and support to two-thirds of the vertebral load and is resistant to mechanical stresses and taphonomic changes due to its strong cortical and dense internal trabecular bone structure [21]. The vertebral column has an intact structure that is less affected by taphonomic changes, yet the spinous and transverse processes are more sensitive to taphonomic alterations and may become fragmented [15, 21]. Therefore, it may be more difficult to collect metric data from these specific landmarks, and further consideration must be taken when developing metric methods from these skeletal elements.

The traditional anthropological assessment of bones consists of the direct observation of skeletal remains [5]. In the past two decades, CT and three-dimensional (3D) reconstruction have played an important role in an increasing number of forensic cases and mass disasters due to the potential application of medical imaging to forensic anthropology [22]. Research has also been conducted in contemporary populations to test and review traditional anthropological methods and generate population-specific data [1, 23].

To provide accurate forensic anthropological information for the identification of unknown individuals, the methods used in the identification process must be tested for error rates [24]. It is well known that among the wide range of techniques used, metric analysis is considered less subjective than morphological approaches, as it is subject to statistical analysis [6]. Moreover, population-specific methods have shown higher accuracies when applied to target populations closer to the reference population. The current study aspires to develop sex estimation equations based on cervical vertebral dimensions in modern Turkish sample employing data from Computed Tomography (CT). This is the first study on the subject in this population.

It is important that the methods and techniques used in forensic anthropology are reproducible and highly reliable [2, 3]. In this respect, a method must be consistently reproducible. Less than 10% errors can be accepted in intra and inter-observer error analysis [2–5]. In this study, intra and inter-observer error tests were applied and showed that there were less than 10% error variations for all intra-observer errors. In inter-observer errors, it showed that there were less than 10% error variations for all other measurements except C3HT and C5AP (*R* = 0.89) (Table 1). Rozendaal et al. [15], in which similar measurement parameters were evaluated, reported error variations of less than 10% for both the Athens and Luis Lopez collections. However, it was reported that the inter-observer error was detected 25.18% for C1TR, which may be due to the misunderstanding of the measurement technique by a researcher. Acceptable error values detected in both studies support that the measured values for the cervical vertebra are reproducible. However, the difference in measurement techniques should be considered. Past studies have shown a high similarity between direct measurements of dry bone and measurements from radiological images of the same bone [25, 26]. Radiological methods are suitable, and CT images in particular can be used in anthropological practice [27]. In addition to CT images, image analysis programs are becoming more common and can contribute to the speed and accuracy in the analysis. Furthermore, the use of radiological images can help address ethical and cultural concerns in situations such as the need to perform maceration involving studies of human remains. Another important point is that medical imaging provides time-saving and rapid evaluation as well as archiving opportunities, especially for mass disaster incidents, including the identification and assessment of trauma. In terms of forensic anthropology, virtual population-specific databases might provide the opportunity for researchers to evaluate, validate and develop methods when osteological collections are not available, with retrospective examination of radiological images assisting in increasing the number of cases that can be examined.

For the purpose of the study, three measurements (CHT, CTR, and CAP) were obtained from each vertebra and all showed significant differences between females and males (*p* < 0.001). The sexual dimorphism indicator (SDI) for the Turkish population was found to be above 10% for the cervical vertebrae maximum body height (CHT) measurements of the vertebrae (C2–C7), indicating that this parameter is strongly sexually dimorphic. Rozendaal et al. [15] performed a study on cervical vertebrae from the Athens and Lopes skeletal collections testing the same parameters as in the present study on the dry bone using Vernier calipers. CHT was found to be the most dimorphic measurement, followed by cervical vertebral foramen transverse diameter CTR [15]. However, Rozendaal et al. [15] stated that the cervical vertebral foramen anterior-posterior diameter CAP measurements were higher in males than in females, although not to a statistically significant degree, except for those of the C1 vertebrae. Marlow et al. [16] and Wescott [28] stated that the most sexually dimorphic parameters in the C2 vertebra included CHT and CTR and that CHT showed lower sexual dimorphism than CTR. Gama et al. showed that the CAP measurement for the C2 cervical vertebra was not significantly different between the sexes, providing a sexual dimorphism index of 2.7 [9]. In the present research, the CTR measurement of the C2 cervical vertebra was not included in the M2 model because it did not make a significant contribution to sex prediction. Amores et al. obtained eight measurements—with the exception of CHT—from the seven vertebrae, with the anterior and posterior distance of the vertebral canal being the most sexually dimorphic and the anterior-posterior distance showing a higher degree of sexual dimorphism than the transverse width [14]. Kibii et al. reported that the centrum in the seventh vertebra shows a higher degree of sexual dimorphism than the vertebral canal and that the vertebral canal CAP and CTR measurements may not show sexual dimorphism in different populations [13], as corroborated in our study, in which C7AP and C1AP demonstrated sexual dimorphism indexes of 8.40% 8.28%, respectively.

Vertebral body heights reach full skeletal maturity in the 20^th^ year of life and thus are more affected by secondary sexual development and environmental effects than vertebral foramen measurements [15, 29]. The CHT measurement shows a higher degree of sexual dimorphism in all cervical vertebrae than the CTR and CAP parameters due to differences in the developmental stages of CHT [15]. Some studies have reported CAP measurements showing minimal sexual dimorphism [15, 30], although other studies have indicated that CAP measurements demonstrate different degrees of sexual dimorphism when assessed in different populations [28, 31, 32]. In our study, sexual dimorphism was indeed observed in the CAP measurements. The sexually dimorphic aspect of the base of the skull would affect the morphological structure of the C1 vertebra because of the association between the two structures [15, 28, 31]. This relationship may affect CAP measurement in the C1 cervical vertebra explaining the sexual dimorphism reported here [15].

The CHT, CTR, and CAP measurements were evaluated together, and logistic regression analysis was performed for each cervical vertebra. The accuracy increased from 83.8% in the C5 vertebrae to 87.60% in the C2 vertebrae. On the other hand, the accuracy in the C1 vertebrae was estimated to be less than 80% [75%]. Andrew et al. stated that no cervical vertebra alone can exceed 80% accuracy for sex estimation [15].

Given the articulation between the cervical vertebrae, when two consecutive cervical vertebrae were evaluated together (LR models M8-M13), the accuracy ranged between 86.70 and 88.10%, and was found greater than those of the single-variable models. Rozendaal et al. combined both the C1 and C2 vertebrae and showed an accuracy of 72.8%; however, C2 and C5 showed an increase in correct classification, resulting in an accuracy of up to 77% [15]. Furthermore, when three consecutive vertebrae were evaluated together in our study, there was no remarkable increase in accuracy relative to that of models generated with two consecutive vertebrae.

In our study, CHT demonstrated the highest degree of sexual dimorphism, and the measurements from all cervical vertebrae were included into a model by stepwise logistic regression analysis (model 14); the resulting accuracy was 87.60%. Additionally, all CTR measurements were included in model 15, yielding an accuracy of 90%. The highest correct classification achieved in this study was 91.40%, which was obtained with a model including five measurements selected by stepwise LR from all cervical vertebrae (model 16). The Rozendaal et al. statistical model included the twenty CHT, CTR, and CAP measurements in total from all cervical vertebrae and yielded an accuracy of 84.1% [15]. When they used stepwise method, seven measurements (C1AP, C2HT, C2TR, C3HT, C5TR, C5HT, C7TR) exhibited large *t*-value coefficients and the discriminant function resulted in accuracy rate of 82.6%. In our model 16, it was striking that there was no CTR measurement. As expected, higher accuracy is obtained by using more measurement parameters and more vertebrae [11, 15, 16, 28].

Along with the dynamics of pathological processes and aging processes in the vertebrae, vertebral anatomy can be directly affected. Degenerative processes in the cervical spine are usually presented in an idiopathic form where no predisposing factor is obvious or a congenital form in which a predisposing factor such as a metabolic disorder and trauma is present. The idiopathic form is related to aging, and the aging process in the cervical spine can cause numerous pathologies involving both surrounding tissue and bone structure [33–37].

Ezra et al. [37] reported that while cervical vertebrae height decreases with age (mainly in C3–C6), the vertebral body expands, vertebral foramen size is independent of age, and emphasized that the enlargement of vertebral bodies may be a secondary mechanism to a decrease in vertebral body height with age. In our study, a difference was found for more parameters in females than in their male counterparts. In the interpretation of the obtained data in relation to age differences, biological and genetic factors, pathological processes and population-specific features related to sex that can affect vertebral dimensions should be revealed in more detail in further research. In this respect, before analyzing the direct effect of the data obtained in our study on sex estimation, it is necessary to consider all these factors in more detail and prospective studies with a better clinical and socio-economic history can be conducted in future studies with an equal and homogeneous age distribution between sexes. As a final note, despite the evidence that the vertebral body exhibits age related changes, introducing the age factor in sex estimation formulae has little practical significance. In essence, if sex needs to be estimated from the vertebrae, this means that accurate age estimation would also be difficult to be performed on the same set of human remains, thus, making the application of age-specific sex estimation formulae impossible. Thus, one may consider using the method, taking into account this inherited drawback, a certain bias on the sex estimate due to the effect of age.

Many studies have demonstrated the impact of sex estimation formulae on skeletal remains that are not closely related to the reference population [4, 6, 15, 32]. Genetics, environmental effects, socio-economic status, and secular differences, among others, are known to affect the size and structure of the skeletal system. Thus, differences between the data of our study and those of other studies are expected based on the aforementioned factors.

Although further validation is required to test the direct applicability of virtual methods on dry skeletal elements and to validate the application of the present formulae to other populations, our research presents the first sex assessment method performed through 3D images of cervical vertebrae in this population and constitutes a contribution to the further development of Turkish population-specific standards.
